# Say *What?* Real-time Linguistic Guidance Supports Novices in Writing Utterances for Conversational Agent Training

**DOI:** 10.1145/3640794.3665554

**Published:** 2024-07-08

**Authors:** Rachel Ostrand, Kristina Brimijoin, David Piorkowski, Jessica He, Erica Albert, Stephanie Houde

**Affiliations:** IBM Research, Yorktown Heights, NY, USA; IBM Research, Yorktown Heights, NY, USA; IBM Research, Yorktown Heights, NY, USA; IBM Research Seattle, WA, USA; IBM, Denver, CO, USA; IBM Research, Cambridge, MA, USA

**Keywords:** conversational agents, utterance design, AI model development, human-computer interaction, feedback

## Abstract

Writing utterances to train conversational agents can be a challenging and time-consuming task, and usually requires substantial expertise, meaning that novices face a steep learning curve. We investigated whether novices could be guided to produce utterances that adhere to best practices via an intervention of real-time linguistic feedback. We conducted a user study in which participants were tasked with writing training utterances for a particular topic (*intent*) for a conversational agent. Participants received one of two types of linguistic guidance in real-time to shape their utterance-writing: (1) feedback on the lexical and syntactic properties and the variety of each utterance, or (2) sample utterances written by other users, to select or inspire the writing of new utterances. All participants also completed a control condition, in which they wrote utterances for a different intent without receiving any guidance. We investigated whether linguistic properties of the utterances differed as a function of whether the participant had received guidance, and if so, which type. Results showed that participants wrote longer and better quality utterances, with greater lexical and syntactic diversity, in both guidance conditions compared to when they received no guidance. These results demonstrate that giving novices explicit linguistic guidance can improve the quality of the training utterances they write, suggesting that this could be an effective way of getting new utterance writers started with much less training than most current practices require.

## 1 INTRODUCTION

Conversational agents, or chatbots, have powered text-based business support for some time, and remain a powerful tool in the space of business automation and customer support [1]. Although there has been a recent explosion of chat interfaces powered by generative AI and large language models (LLMs), traditional conversational agents remain an important and widely-used tool, as they do not require the vast amounts of training data, compute power, or training time of LLMs, and can be initialized with a relatively small number of training examples. In addition, traditional conversational agents produce consistent and predictable responses to users’ questions and requests [12], and are not susceptible to “hallucinations” or potentially producing harmful, biased, or false responses – problems which affiict LLM output [7, 33, 40, 47]. Such behavior is particularly important for business applications in heavily-regulated industries, such as the financial or healthcare sectors, or other critical domains in which an automated agent must be able to deliver a precise, known response consistently [12]. However, the downside of this benefit is that conversational agents require a much more focused dataset for initial training in comparison to that required for LLMs; in particular, they require a range of example text inputs (known as *utterances*) that comprise different ways to phrase the same meaning or topic (known as *intents*). Writing these initial utterances is time-consuming and generally requires experienced utterance writers. In the present work, we investigate whether it is possible to guide novices who have knowledge about the *topic* in which the conversational assistant operates (e.g., banking or travel booking) but no *utterance-writing* experience to write utterances that adhere to best-practices, thus avoiding the current substantial start-up costs of training a new expert utterance writer. To do so, we ran a user study in which novices interacted with a tool that gave them real-time linguistic guidance as they wrote new utterances for a conversational agent. Different participants received different types of guidance, enabling us to investigate the linguistic differences that emerged in the utterances across the different types of guidance support.

## 2 BACKGROUND AND RELATED WORK

### 2.1 Challenges of Writing Training Utterances

Arguably the most challenging part of training a new conversational agent is acquiring sufficient training utterances. This is one of the few aspects for which initialization of a conversational agent is more time- and effort-intensive than for a large language model; although an LLM requires much *more* training data and substantially more time and computational power to train, the data does not need to be labelled or curated [20, 38]. Fortunately, developing training utterances for a new conversational agent is a one-time process; once an agent has been deployed to users in production, then real user utterances from conversation logs can be used for further training and refinement of the agent.

But writing those initial utterances is often challenging and expensive [2, 39, 45]. It can be difficult for a developer to predict how end users will verbalize their intent, particularly for applications with an international user base and users who speak a variety of languages or dialects [42], as utterances can be highly language- and domain-specific [14]. A conversational agent’s success at identifying the users’ requests depends on having high quality, varied training utterances which accurately map to the appropriate intent, so the quality of these initial utterances can have a major effect on how successful, and thus useful, an agent is upon initial deployment [44]. Many commercial systems provide some support for this step (e.g., IBM’s watsonx Assistant includes a feature for recommending intents and synonyms [4]), but much of it falls to human experts who have been trained in the art of crafting intents and utterances for conversational agents. This training process often requires several iterations of re-training once the conversational agent has been deployed [5]. Thus, an important question arises of how best to guide people to write good training utterances for the initial deployment of a conversational agent, and what that guidance should consist of.

### 2.2 Design of Traditional Conversational Agents

Traditional conversational agents vary in the specifics of how they work, but share a similar conceptual design. Generally, they can be separated into two parts: intent recognition and dialogue logic. The intent recognition maps the end user’s text utterance to an intent that the conversational agent has been trained on for that conversational turn. There are many possible utterances that could all map to the same intent. For example, an intent of “fetch room rate” for a hotel conversational agent might be detected from the various utterances of “How much does a room cost?” or “Tell me the hotel nightly rate” or “What is the price for a king bed suite?” Some intents are general (e.g., greetings), but most are aligned to the specific functionality of the agent, such as a hotel agent trained to detect intents about a hotel’s rates, services, and room availability. The recognized intent is then used by the dialogue logic which defines the flow of conversation depending on the intent, collects relevant data from the user, and interacts with other services to provide the agent’s functionality.

A conversational agent can only recognize the specific intents that it was trained on. Machine learning classifiers are typically trained using multiple example utterances for each intent, so that when the conversational agent receives a new, unseen utterance from a user, it can predict which intent it maps onto.

Traditional conversational agents are inherently more linguistically and functionally limited than conversational agents trained with LLMs. However, for some use cases, this limitation is preferable. Some domains such as finance and insurance desire oversight for agent output, and the output of a traditional agent is fixed, as it is defined in the dialogue knowledge. In contrast, an LLM-based model’s generative nature makes it more difficult to predict what the agent might say to a user. In particular, hallucinations, in which an LLM provides false information, pose a risk to conversational agent owners and remain an unsolved problem [27]. Until such risks can be fully mitigated, traditional conversational agents remain a relevant and necessary tool for business interactions with users and clients.

### 2.3 Support for Developing New Training Utterances

There is little prior work that investigates ways to facilitate writing effective conversational agent training utterances. One study looked at training non-experts to write prompts for an LLM system with the support of an automated tool, which gave guidance both for authoring prompts and labelling errors in the conversation [46]. However, this study focused on writing input to an LLM rather than a traditional conversational agent, and also on improving an existing bot rather than creating a new bot from scratch. Another study focused on developing *ideas* for how users can improve the generated text output from a model [10], but did not address the implementation of tools or types of feedback to support users in writing these utterances. Techniques exist for automatically identifying intents [17, 31, 32, 41] and enabling more interactive training and assessment of agents [43], but these techniques require pre-existing utterance log data. Thus, integrating utterance-writing guidance into writers’ tooling, especially if targeted at reducing the burdens of onboarding novices, is an important gap in the research and technology space for developing conversational agents.

Prior research in other domains and tools has shown that users are willing and able to take advantage of automated support. *GuiComp*, a tool to assist novice designers in the creation of graphical user interfaces, reduced user effort and produced better designs despite the targeted users having little to no design experience [29]. In a study of *HelpMeOut*, a plugin for Integrated Development Environments that collects and provides solutions to common compiler and runtime errors, novice programmers received helpful solutions 47% of the time [22]. *iChatProfile*, an assistive tool for creating interview chatbots that uses transcripts to generate a chatbot profile and offers performance metrics and design suggestions, helped users improve their chatbot’s response quality, user engagement and user experience [21]. Finally, in a study investigating interactive dialogue with a conversational agent, users tailored their utterances and repeated more of the agent’s own words when the agent gave them real-time feedback about its poor comprehension ability [35]. These demonstrations of automated feedback from a conversational agent to a user suggest that this may be an effective tool in the utterance-writing domain as well.

The goal of developing tools to support utterance writers to write “better” training utterances raises the question of what linguistic properties utterance-writing experts strive for when training a new conversational agent. We are aware of only one study that addressed this question, which investigated the work practices of utterance writers and determined that guidance on language tone, semantics, and syntactic elements should be integrated into utterance-writing platforms [8]. Some commercial conversational agent development tools provide best practices for writing training utterances (e.g., [4, 16, 23]), or provide interfaces which support conversational agent training, including aspects of the utterance-writing process. For example, IBM’s watsonx Assistant offers a graphical user interface to create dialog flows and provide examples for intents, a conflict analyzer that flags overlapping utterances and, once a conversational agent is deployed, recommendations for new intents or examples to improve existing intents based on conversation logs [4, 26]. Microsoft Azure AI services offer the ability to define entities in utterances, provide phrase lists, test and make corrections, in addition to dialogue flow management [34]. A differentiating feature of Google’s Dialogflow is a validation tool that the user can invoke to see issues with the intents, utterances, entities or dialog flow [15]. To the best of our knowledge, Dialogflow’s validation feature is the closest approximation to utterance-writing guidance that is embedded in the application interface. While Dialogflow provides utterance-level feedback on similarity between utterances, there remains a gap in exploring other types of best practices to encode into a feedback system and measuring the impact of such features on utterance quality.

With these points in mind, we developed an interactive utterance-writing guidance tool to give novices real-time feedback on their utterances. Then, we ran a large-scale quantitative study to evaluate how well the tool enables novices to write utterances which conform to the best practices that professional utterance writers strive for when crafting a training set for a new conversational agent. Our tool presents two types of utterance-writing guidance, and we assess how well each type of guidance aids novices in their utterance-writing.

### 2.4 Best Practices for Utterance-Writing and Design of Experimental Conditions

To decide on the types of feedback that our utterance-writing tool would provide to users, we consulted documentation for conversational agent development platforms [4, 16, 23] as well as utterance-writing experts whose job roles included conversational agent training and development. The goal was to understand what linguistic properties constitute high-quality training utterances to inform the design of the guidance conditions for the following study.

In reviewing best-practices guidance from utterance-writing experts and conversational agent development documentation, two main themes recurred. First, the guidance emphasized the importance of linguistic variety among a set of utterances to capture the many ways that different users refer to the same intent. In particular, it was noted that syntactic variety — including utterances with a broad range of grammatical structures and phrasing — and lexical variety — varying key content words in the utterance, especially nouns and verbs — are central to writing a set of high-quality utterances. In addition, although the ideal number of words in an utterance varied depending on the agent being trained, there was a range of utterance lengths which hit the sweet spot across conversational assistants – neither very long nor very short.

Second, although such linguistic variety is ideal for writing training utterances, it is challenging to achieve with only a small set of utterance writers, even experts, due to individual and cultural differences among future end users that may not be known until after the conversational agent is deployed. For example, dialectal differences could lead to a situation where US-based utterance writers fail to train the agent on terms that UK- or India-based users produce for the same concept (e.g., “pay raise” vs. “pay increase” vs. “pay hike”), resulting in a comprehension failure by the agent. Such dialectal and cultural differences are challenging to anticipate and write into a training set simply by striving for lexical and syntactic diversity. Instead, to consider dialectical differences, a common strategy is to refer to chat logs which include utterances written by real users of the agent, often spread across geographies and native languages and dialects. Unfortunately, this solution is currently only available for conversational agents that have already been deployed.

These two forms of guidance became the basis of the experimental conditions that we developed and evaluated in the current study: (1) linguistic feedback that alerts writers to lexical and syntactic similarity across their utterances and (2) sample utterances that provide real examples of varied end user language prior to deployment. The goal was to experimentally test whether one or both of these forms of guidance could support novice utterance writers in crafting a set of training utterances that conformed to the desired linguistic properties. The first condition was designed to investigate whether providing automated, utterance-level instructions on increasing linguistic diversity would be an effective method of influencing users’ utterances. The second condition was intended to test whether crowd-sourcing utterances from non-expert users through surveys was a viable way to collect end user language *prior* to deploying a conversational agent, and then providing these crowd-sourced utterances for novice utterance writers to select the most effective ones as a form of iterating on the training set via human input.

## 3 METHODS

### 3.1 Participants

The participants were 187 employees of IBM, a large, multi-national technology company. Data from an additional 19 participants who completed the study were discarded for performance reasons, either because they did not complete the experiment in one sitting as instructed (i.e., took one or more long pauses; 8 participants) or did not provide appropriate utterances for the task (e.g., utterances that did not address the topic of the prompt, or were copied verbatim from the experiment tutorial; 11 participants). Other than being over age 18 and an IBM employee, there were no additional inclusion or exclusion criteria for participation. Of the 187 participants, 94 were assigned to the Language Feedback condition and 93 to the Sample Utterance condition (as described below).

We aimed for a participant sample with varied demographics and experience with the goal of generating a wide variety of training utterances in terms of linguistic usage, topics, dialectal variations, and so on. In particular, because a goal of this study was to investigate whether feedback provided to *non-expert* utterance writers can produce high-quality training utterances when aggregated across participants, we did not recruit for any particular job roles or expertise, and instead recruited widely across regions, job roles, and company divisions. The participant gender distribution was 106 female, 75 male, 6 prefer not to report; with a range of ages: 18–25 years (N=35), 26–35 years (N=51), 36–45 years (N=44), 46–55 years (N=30), 56–65 years (N=23), 66–75 years (N=4).

The experimental tasks were conducted in English. We similarly recruited for a range of English nativeness and proficiency level among participants. Ninety-six participants had been exposed to English from birth, 50 were exposed in early childhood before age seven, and 41 after age seven. Most self-rated their proficiency level as relatively high on a 7-point scale, with 96 rating themselves *7 (native-level proficiency)*, 50 rating themselves *6*, 24 rating themselves *5*, and 17 rating themselves at *4 (medium)* or below. Participants reported a total of 33 other languages that they either were exposed to from birth and/or spoke fluently.

**Figure 1:**
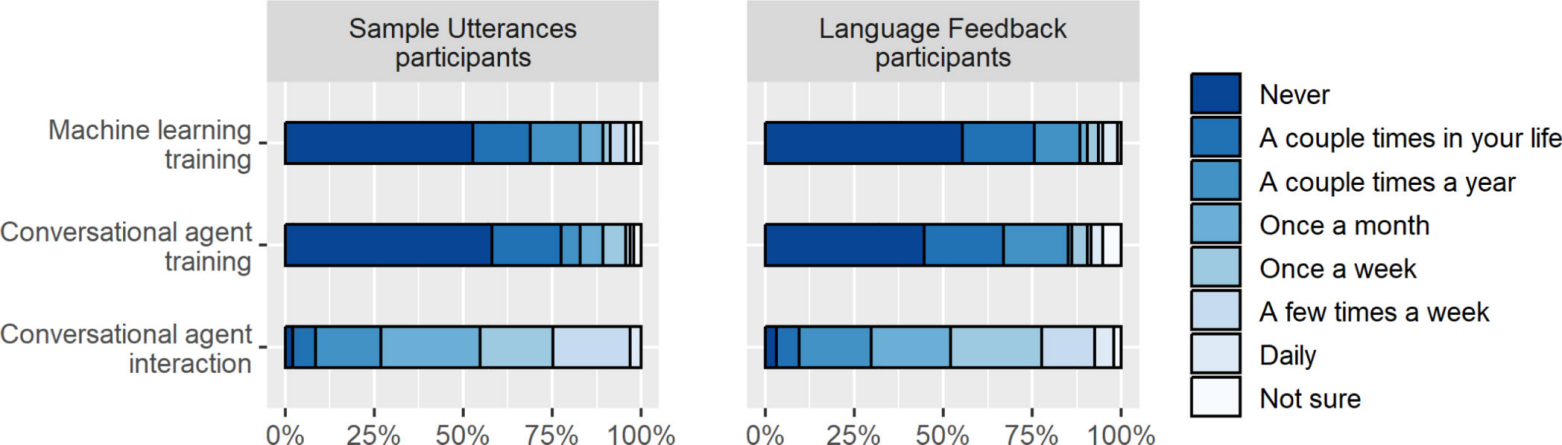
Participant’s self-reported experience with conversational agents and machine learning. Participants rated their experience with: (1) working on building or training machine learning models, (2) working on building or training conversational agents, and (3) interacting with conversational agents in their daily life.

Participants also had a range of experience with conversational agents and machine learning, and rated themselves on a 7-point scale on three metrics: (1) how frequently they worked on building or training machine learning models, (2) how frequently they worked on building or training conversational agents, and (3) how often they interacted with conversational agents in their daily life. The data are shown in Figure 1.

The experiment took approximately 30 minutes to complete, and participants were compensated with company points worth the equivalent of $12.50. All participants provided written informed consent and were treated in accordance with the guidelines for ethical treatment of human participants.

### 3.2 Procedure

Participants interacted with a tool to write training utterances for a conversational agent. The experiment was unmoderated and participants proceeded at their own pace. It began with an overview, which described the experiment scenario and the basics of conversational agents and intent training. Each participant then completed two rounds of utterance-writing. For all participants, the first round was the *Control* condition, in which no guidance on their utterances was provided. The goal of the Control condition was to collect a participant-specific baseline for properties of their utterances. In the second round, each participant received one of two experimental conditions, either *Language Feedback* or *Sample Utterance*; assignment of experimental condition was between-participant. The guidance that was provided to participants as they wrote their utterances differed as a function of condition, as described in the following sections.

In each round, participants were shown one intent (see Table 1) and asked to write at least 10 representative training utterances for that intent. Participants were shown the name of the intent along with a short description of what the intent topic covered. For all three conditions, once the participant had written 10 utterances, the “Submit” button became active and they were able to advance to the next part of the experiment. However, participants were allowed to write more than 10 utterances for each round if they chose.

In the last stage of the experiment, participants completed a post-experiment questionnaire which collected information about their perceptions of the tool, as well as demographics, language history, and level of familiarity with conversational agents and machine learning (as described in Section 3.1).

#### 3.2.1 Control Condition.

All participants began the experiment with the Control condition. The Control condition provided no real-time or utterance-specific guidance. The only information provided in this condition was a static side panel with a list of guidelines reflecting the best practices determined from expert utterance writers and commercial agent documentation (see Section 2.4). This information panel gave high-level suggestions to participants to vary key terms, phrases, and syntactic structures, to avoid filler words, and to include only one request or question in each utterance, but did not give detailed instructions or examples. This panel was intended to simulate documentation pages containing best practices that are currently available to utterance writers.

#### 3.2.2 Language Feedback Condition.

**Figure 2:**
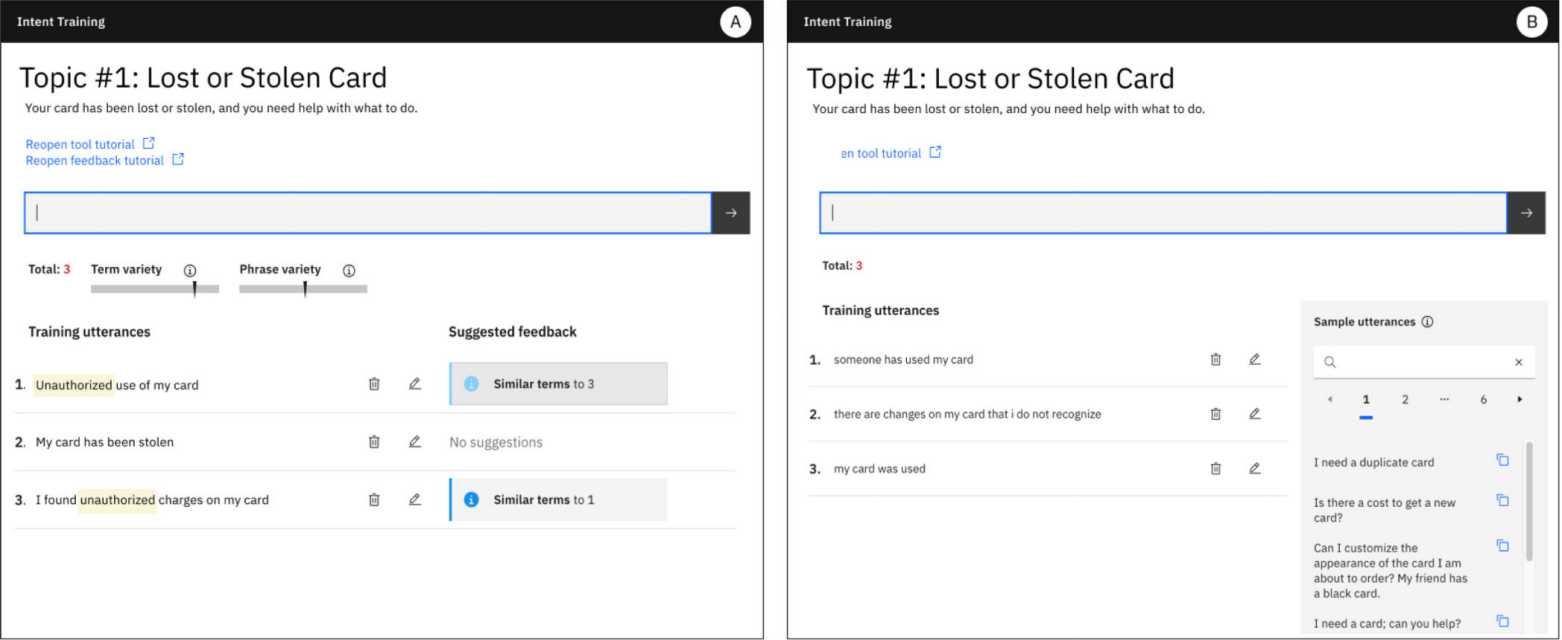
The intent training tool in the two experimental conditions. (A) The interface for the *Language Feedback* condition shows the intent name and description at the top, utterances that have already been written by the user in the utterance editor on the bottom left, and feedback on lexical and syntactic variety for those utterances on the bottom right. (B) The interface for the *Sample Utterance* condition also shows the intent name and description at the top and utterances that the user has already written in the utterance editor on the bottom left, along with a paginated, searchable panel of sample utterances on the bottom right.

In the Language Feedback condition, participants received linguistic feedback on individual utterances as they wrote them (see Figure 2A for a view of the tool interface in this condition). The tool provided several types of feedback based on best practices. Two types of feedback, *Term variety* and *Phrase variety*, were computed across the utterances as a set. These two measures were each displayed as a scale at the top of the utterance-writing panel with a needle showing the computed value of the current set of utterances. The other types of feedback were assessed on each utterance separately, and appeared as a feedback notification adjacent to each individual utterance for which that feedback applied. Hovering the cursor over a notification highlighted the word(s) within the utterance(s) that contributed to that feedback. For example, hovering over the *Similar terms* notification highlighted the similar terms across *all* utterances. The types of feedback are detailed in Table 2.

As the participant added new utterances or modified existing utterances, notifications next to individual utterances updated in real-time. For example, if the user wrote a new utterance that had several words which overlapped with an existing utterance, then both the new and existing utterance would have a *Similar terms* feedback notification appear next to them. Similarly, as the user added, edited, or deleted utterances, the needles showing the value of the *Term variety* and *Phrase variety* scales recalculated and updated. Participants were not required to respond to feedback notifications.

#### 3.2.3 Sample UFerance Condition.

In the Sample Utterance condition, the tool included a panel with a list of approximately 100 sample utterances for the current intent (see Figure 2B). Participants were not required to use these samples, but if they chose to do so, they could search through the list for inspiration, and either directly copy-paste an utterance from the list into their own set of utterances, or draw ideas from the sample utterances to write new ones. This condition gave participants guidance in the ‘wisdom of the crowd’ sense, by allowing participants to check if their ideas about the intent aligned with other users, and to help generate utterance ideas that they may not have thought of on their own.

These sample utterances were derived from a crowd-sourcing pre-survey in which 20 participants provided examples of how they would “ask the chatbot to help you” for a given intent. Participants in this preliminary survey wrote 5–10 utterances for each of the four intents used in the main experiment, resulting in a total of 433 sample utterances across the four intents (range: 105–112 utterances per intent across participants; 20–34 utterances written per participant). Study participants were drawn from the same study population as those in the main experiment (i.e., IBM employees), but consisted of a distinct sample of individuals.

### 3.3 Experimental Design

There were four intents utilized across participants in the experiment. Each participant wrote utterances for two of the four intents, one for the Control Condition and a different one for the Experimental Condition. The intents were selected from the openly-available Banking77 dataset, a collection of 13,083 real banking customer service interactions that have been categorized into 77 intents [9]. The four intents related to basic bank card interactions (e.g., your card was lost or stolen), and were selected as topics that were likely to be familiar to participants from their personal experience, and thus did not require them to have specialized knowledge. See Table 1 for the four intents and two example utterances for each. The motivation for choosing four intents to counterbalance across participants was to reduce any possible effects of preference, personal knowledge, or other forms of bias by participants. For example, if participants had more personal experience for one of the intents, then that could lead them to write more varied and a greater number of utterances for that intent, irrespective of the guidance provided. By randomly selecting from four intents across participants, we reduced the impact of specific features of any one intent on the observed results, allowing for greater confidence in their generalizability to unstudied intents or topics.

Assignment of experimental condition (*Language Feedback* vs. *Sample Utterance*) and intent in each of the control and experimental conditions was counterbalanced across participants, for a total of 24 unique combinations (2 Experimental conditions x 4 possible intents for control x 3 possible intents for experimental). Due to participants who launched the tool but did not complete the study, the number of participants varied slightly across combinations of intents and experimental conditions. However, with the large sample size, the number of participants in each set was relatively balanced, with 19–28 participants in each combination of experimental condition and intent.

## 4 RESULTS

This experiment investigated whether and in which ways participants modified the linguistic characteristics of their utterances as a function of the guidance they received. As the goal of our tool is to help users write better utterances, the purpose of the current work was to measure the ways in which these utterances themselves changed. To do so, we ran two types of analyses. The first measured key lexical properties of the utterances. The second assessed how well the linguistic content of the utterances followed utterance-writing best practices guidelines.

### 4.1 Analysis Methods

The analyses were conducted using generalized linear mixed-effects models in R (version 3.6.2) [37] using the *lme4* package (version 1.1.21) [3]. The statistical models were the same for all analyses described in the following sections, except for the dependent variable, as described below. Each omnibus model included two categorical independent variables: Condition (Control vs. Experimental; within-participants) and Guidance Type (Language Feedback vs. Sample Utterance; between-participants). Both independent variables were sum-coded. The dependent variables were all continuous. Test statistics and statistical significance for each effect were determined using the *lmerTest* (version 3.1.2) [28] and *emmeans* (version 1.5.0) [30] packages, employing Satterthwaite’s method for approximating degrees of freedom, with a significance threshold set at .05 for all statistical tests. Following the omnibus model, pairwise contrasts were conducted to explore comparisons between each level, with *p* values adjusted for multiple comparisons using the Tukey correction. Note that although the statistical models were conducted in log-odds space, the figures and condition means reported in the text show untransformed data, as this scale makes the interpretation of effect sizes easier. The data and code for running the analyses detailed below are available in the following repository: https://osf.io/e9j8p/.

### 4.2 Lexical Features of Participants’ Utterances

**Figure 3:**
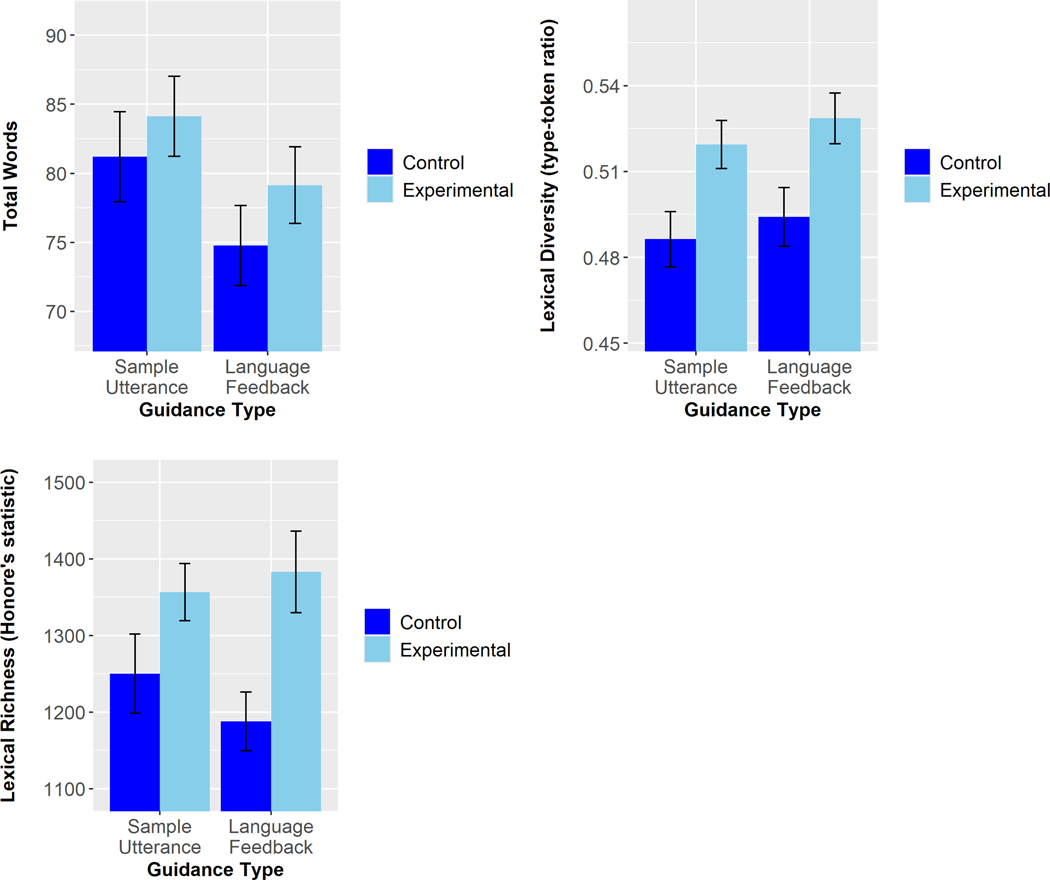
Lexical features of participants’ utterances: (A) Total words, (B) Lexical diversity, (C) Lexical richness, as computed on the utterances written as a function of Guidance Type and Condition. Error bars show standard error of the mean.

The goal of these analyses was to understand how participants changed the linguistic content of their utterances as a function of the guidance that they received. These lexical features were computed automatically using Python on the punctuation-stripped utterances written by participants. The results are presented graphically in Figure 3.

**Total word count** was investigated as a proxy for participant engagement with the utterance-writing task, on the theory that more words written – either via additional discrete utterances or more detail in each utterance – would be expected if the experimental conditions were successful at providing guidance (cf. [24]). In the omnibus model, there was a numerical difference but not statistically significant effect of Condition (*t* = 1.935, *p* = .055), such that participants wrote more words across utterances in the Experimental condition (mean = 81.6 words) compared to the Control condition (mean = 78.0 words). There was also no main effect of Guidance Type (|*t*| < 1.6), and no interaction (|*t*| < 1), and none of the pairwise comparisons reached significance (all |*t*| < 1.7, all *p* > .11).

**Lexical diversity** was investigated as a way to measure the degree to which participants wrote a range of distinct words, as opposed to repeating the same words within and across utterances. This is an important property to optimize when writing training utterances for conversational assistants and dialogue systems, given how varied natural language is [39, 45]. Lexical diversity was computed using type-token ratio, the ratio of the count of different words produced (types) to the total words produced (tokens), with higher values indicating more varied word choice. Note that this measure is related to, but different than, lexical similarity (discussed below in Section 4.3), in that lexical diversity measures the ratio of *exact word repetitions* both within and between utterances, whereas lexical similarity measures the semantic (content) similarity between (but not within) utterances, which may be high even with zero repeated words. Greater lexical diversity is expected if the experimental conditions were able to provide useful guidance, by producing utterances which are more natural [24].

There was a significant main effect of Condition (*t* = 4.638, *p* < .001): Utterances in the Experimental condition (mean = 0.524) had greater lexical diversity than utterances in the Control condition (mean = 0.490). There was no main effect of Guidance Type and no interaction (both |*t*| < 1). The pairwise comparisons supported the main effect of Condition, such that in the Sample Utterance condition, the Experimental condition elicited higher lexical diversity than did the Control condition (*t* = 3.205, *p* = .009) and in the Language Feedback condition, the Experimental condition elicited higher lexical diversity than did the Control condition (*t* = 3.355, *p* = .006).

**Lexical richness** was investigated as deeper method of measuring how varied the word choice of participants’ utterances was. It was computed using Honoré’s Statistic, a measure which focuses on the number of words within a text which are produced exactly once (relative to the types and tokens in the text), with higher values indicating richer language [25]. This measure captures the long tail of the distribution of word choice in the utterances, and has been used to detect (e.g.) early-stage cognitive decline in speakers, demonstrating its utility for capturing the richness of language [6, 19, 36]. There was again a significant main effect of Condition (*t* = 3.883, *p* < .001), such that utterances in the Experimental condition (mean = 1370) showed greater lexical richness than did utterances in the Control condition (mean = 1219). Again, there was no main effect of Guidance Type and no interaction (both |*t*| < 1.2). In the pairwise comparisons, the Control-Language Feedback condition elicited significantly *less* rich speech than both the Experimental-Language Feedback condition (*t* = 3.565, *p* = .003) and the Experimental-Sample Utterance condition (*t* = 2.604, *p* = .047).

### 4.3 Comparison of Properties of Participants’ Utterances to Best Practices

**Figure 4:**
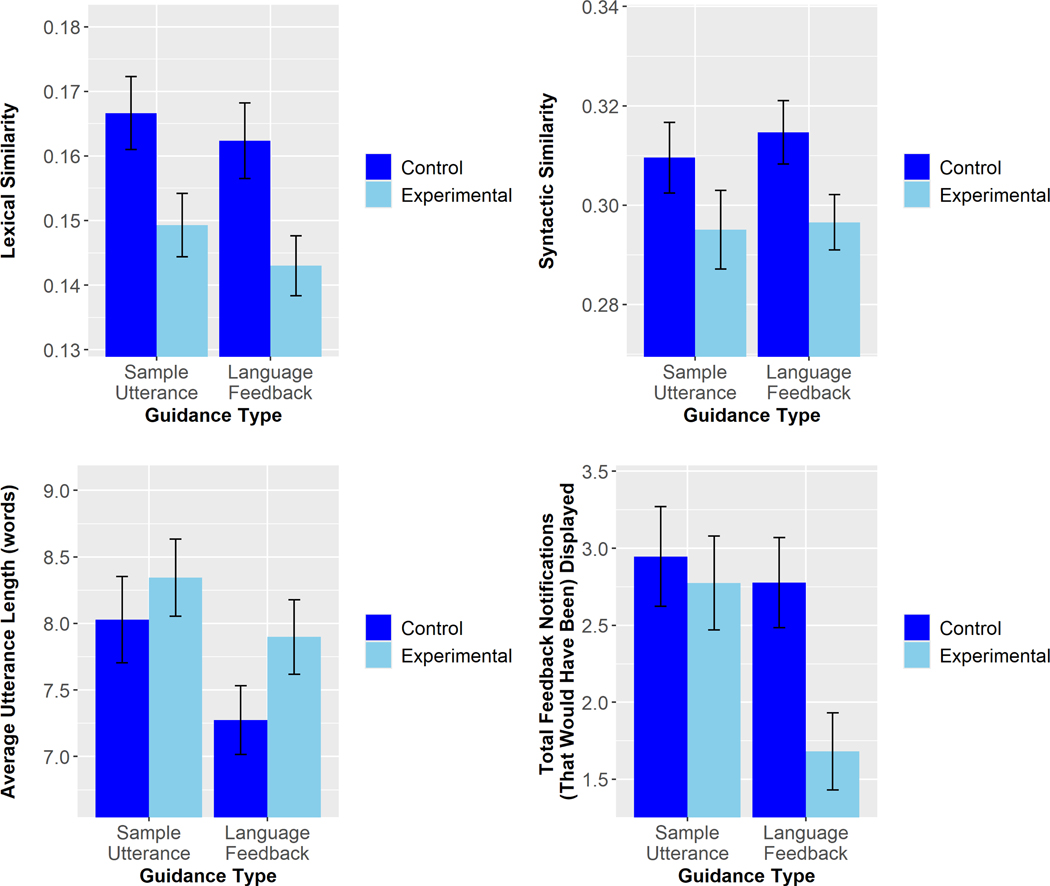
Best practices features of participants’ utterances: (A) Lexical similarity, (B) Syntactic similarity, (C) Utterance Length, (D) Total number of feedback notifications, as computed on the utterances written, as a function of Guidance Type and Condition. Error bars show standard error of the mean.

The goal of this set of analyses was to investigate how well participants’ utterances adhered to industry best practices. Four features were computed: (A) lexical similarity, (B) syntactic similarity, (C) average utterance length, and (D) total number of utterance-specific feedback notifications that *would* be displayed for the participant’s final set of submitted utterances. The results from these analyses are shown in Figure 4.

**Lexical similarity** was computed as follows. First, we calculated the TF-IDF (term frequency, inverse document frequency) vector for each training utterance against all of a participant’s submitted training utterances. Prior to the computation, stop words were removed and all words were stemmed using the Porter Stemmer algorithm ^1^. We then calculated the cosine similarity of each pair of utterance TF-IDF vectors, resulting in a value that ranged from 0 (completely dissimilar) to 1 (completely similar). Any pair of utterances with a cosine similarity greater than 0.7 was considered similar and would result in a feedback notification. This threshold was determined via pre-experiment testing: The authors tested the tool with the notification threshold set at various levels, and selected a threshold such that a similarity notification would appear when the generated utterances seemed fairly overlapping, and a notification would not appear when the utterances seemed mostly dissimilar. This was, therefore, a subjective rather than empirically-driven decision; however, we do not claim that this threshold is the ideal one for providing a similarity notification (as we did not compare multiple feedback notification thresholds), and it is possible that setting the notification threshold at a different value would have resulted in slightly different levels of utterance lexical similarity. Importantly, because our results compare linguistic behavior across multiple conditions, what matters is the *relative* lexical similarity across conditions, not the absolute similarity score in any individual condition. As per the best practices guidance, more effective utterances should have *lower* lexical similarity across utterances.

There was a significant main effect of Condition (*t* = −4.816, *p* < .001), such that utterances in the Experimental condition had lower lexical similarity (mean = 0.146) than did utterances in the Control condition (mean = 0.164). There was no main effect of Guidance Type and no interaction (both |*t*| < 1). The effect of lower lexical similarity for the Experimental condition was significant in the pairwise comparisons as well, with utterances in the Experimental-Sample Utterance condition displaying lower lexical similarity than those in the Control-Sample Utterance condition (*t* = 3.210, *p* = .008), and utterances in the Experimental-Language Feedback condition displaying lower lexical similarity than those both in the Control-Language Feedback condition (*t* = 3.602, *p* = .002) as well as the Control-Sample Utterance condition (*t* = 3.156, *p* = .010).

**Syntactic similarity** was computed as follows. Each utterance was tagged for part of speech (POS) using Brill’s POS tagger ^2^, creating a sequence of POS tags for each utterance. We then compared each pair of utterances for the longest common sub-sequence of matching tags. If the ratio of sub-sequence length to the utterance length was greater than 0.6, the pair of utterances was counted as similar and would display a feedback notification (note that the possible range of values is 0 to 1). As with the lexical similarity notification threshold, the syntactic similarity notification threshold was determined via the authors’ testing, and set at a level that seemed a good dividing line between similarly-structured utterances inducing a notification and largely different utterances not inducing a notification. Again, as per best practices guidance, more effective utterances should have *lower* syntactic similarity.

In the omnibus model, there was again a significant main effect of Condition (*t* = −3.401, *p* < .001), such that utterances in the Experimental condition had lower syntactic similarity (mean = 0.296) than did utterances in the Control condition (mean = 0.312). There was no main effect of Guidance Type and no interaction (both |*t*| < 1). Of the paired contrasts, the Experimental-Language Feedback condition showed lower syntactic similarity than did the Control-Language Feedback condition (*t* = −2.677, *p* = .040).

**Average utterance length** investigated the best practice guidance of avoiding overly long or overly short utterances. A feedback notification was triggered for any utterance with a length greater than 17 words; this threshold was determined by selecting the 85th percentile of utterance length in the Banking77 [9] dataset.

In the omnibus model, there was a significant main effect of Condition (*t* = 2.653, *p* = .009), such that utterances were longer in the Experimental condition (mean = 8.12 words) than in the Control condition (mean = 7.65 words). As the average utterance length in both conditions was substantially below the threshold of 17 words to trigger a feedback notification, this result likely reflects participants writing more detailed utterances when they received guidance. There was no main effect of Guidance Condition (|*t*| < 1.6) or interaction (|*t*| < 1). The only paired contrast that reached significance was that of the condition with the shortest utterances (Control-Language Feedback) compared to that with the longest utterances (Experimental-Sample Utterance; *t* = 2.616, *p* = .046).

The final measure was the **total number of feedback notifications** that would have been displayed to participants based on their full set of submitted utterances. In the Experimental-Language Feedback condition, these notifications were actually shown, and participants had the opportunity to modify their utterances to address the specific feedback, but in the other three conditions, the specific feedback notifications were not shown to participants. Therefore, this analysis investigated how well participants tailored their utterances holistically to the best practices guidelines – either because they received specific feedback notifications, used the sample utterances provided, or learned from the high-level instructions. There was a significant main effect of Condition (*t* = −2.602, *p* = .010), such that utterances in the Experimental condition elicited fewer notifications (mean = 2.225) than did utterances in the Control condition (mean = 2.861). There was no main effect of Guidance Type (*t* = −1.876, *p* = .062) or interaction (*t* = −1.896, *p* < .060). However, the paired contrasts revealed differences between individual conditions, with the utterances in the Experimental-Language Feedback condition eliciting significantly fewer notifications than did utterances in each of the other three conditions (all |*t*| > 2.632, all *p* < .044).

## 5 DISCUSSION

This experiment investigated whether novices could be tapped to write effective training utterances for a conversational agent, work that traditionally has relied on human experts who have undergone substantial training. We found that providing novices with real-time linguistic guidance can have a meaningful influence on the training utterances that they wrote; in particular, guidance improved linguistic characteristics that bring the utterances more in line with best practices in the industry, compared to the utterances that novices wrote without receiving interactive guidance. This suggests that such guidance could be a fruitful method of obtaining a large set of quality training utterances, especially when utterance-writing experts are not available or are in short supply, such as for a conversational agent in a new domain or for a new company. Additionally, providing this type of real-time guidance to utterance writers might allow an agent to be deployed with a smaller set of training utterances if they were of higher-quality.

One surprising result that emerged from the current study was that both types of guidance were equally effective at influencing participants’ utterance-writing, and resulted in equivalently improved utterances on several metrics, including higher lexical diversity and lower lexical similarity, lower syntactic similarity, and longer or more detailed utterances. This suggests that a viable strategy for including novices in the utterance-writing process could take different forms depending on what resources are available to a conversational agent developer.

### 5.1 Implementation in Practice

Although the current study was conducted in a controlled environment, the methods tested here could be deployed by conversational agent developers fairly easily. One possibility would be for developers to crowd-source an initial, unfiltered set of example utterances – either using internal employees, naive participants via a crowd-sourcing platform such as Amazon’s Mechanical Turk, or automatically via natural language generation – and then employing a separate set of participants to refine this initial set of utterances into higher-quality utterances for training before deployment, mimicking the *Sample Utterance* condition of the present study. This process of selecting and iterating on the set of training utterances could even be conducted multiple times across multiple unique groups of participants, for further refinement of utterance topics and wording, drawing upon the “wisdom of the crowd” [13] and people’s disparate linguistic and cultural experiences to cover a broad range of phrasing, word choice, and intent conceptualization. This method of utterance generation and refinement is probably the most useful for conversational agents that operate in a domain for which regular people without any special training or expertise have personal experience, allowing them to write training utterances which approximate those written by eventual end-users. The domain tested in the present experiment – common situations involving a bank card – was selected for precisely this reason. Although the particular training utterances generated in the present study are domain-specific, similar utterances could be generated using this iterative process for any domain for which non-expert users are likely to be familiar.

Alternatively, if multiple sets of novice utterance writers or refiners are not available, or if the conversational agent’s domain is one which requires more specialized expertise than a layperson has, then providing real-time linguistic guidance within an interactive tool could be similarly effective, following the *Language Feedback* condition. For example, a conversational agent developer could recruit participants who are subject-matter experts in the *domain* for which the conversational agent operates (e.g., income tax law [11, 18]), but it would not be necessary to restrict the participant pool to those who have been trained in *utterance-writing*. The tax professionals could write training utterances for the tax conversational agent, and then refine them using the linguistic feedback provided in our tool, allowing for a larger pool of utterance writers and thus more linguistic diversity among the training utterances.

### 5.2 Limitations and Future Directions

There are a few factors that might limit the generalizability of our conclusions to other systems and users. First, although we made a concerted effort to recruit a diversity of participants across native languages, geographies, job roles, and experience with conversational agents, all of our participants had one important characteristic in common – they were employees of a large business technology company, and thus likely had a higher baseline level of exposure to conversational agents and related technologies than does the average layperson. As a result, our participants’ utterances might be higher quality than those produced by more technology-naïve writers (although the experimental design of comparing an individual’s utterances in a no-guidance vs. a guidance condition allows us to explore the *change* in quality within-participant). This suggests an important follow-up for future research: testing a participant sample which is not immersed in a business technology environment, to see if the feedback mechanisms are helpful even for more technology-naive utterance writers.

Second, our study was conducted in a sandboxed environment, and was not tested on a real conversational agent deployed in the field. It is possible that, although the utterances written in our experiment had linguistic properties which closely aligned to those targeted by industry best practices, they would nevertheless not be successful in training a conversational agent. Thus, a second important direction to explore in future work would be to take the utterances generated by novices using the feedback mechanisms outlined in the current study, train a real conversational agent, and compare that agent’s performance against an agent which was trained exclusively on expert-written training utterances. If performance is comparable, or within an acceptable margin, that would provide more support for employing the utterance-writing feedback techniques discussed here.

On a related note, this study measured how well the utterances fit with certain linguistic metrics, but did not evaluate how well the content of the utterances fit with the intended intent. It is certainly possible to write an utterance which scores well on the measured linguistic features but has nothing to do with, say, ordering a new bank card. Similarly, an utterance might be relevant to the intended intent, and *also* a completely different intent, and thus be a poor training example. Such assessments are beyond the scope of the present work but remain an important direction for follow-up research. An utterance’s relevance could be quantified by computing a semantic similarity metric between each new utterance and existing utterances, or with a clustering algorithm to measure how well the utterances intended for each intent group together. In the current study, the Sample Utterances condition provides a rough way of measuring utterance relevance, as the participants presumably selected or refined utterances from the list of samples that were most relevant to the intended intent.

In addition, prior work found that expert utterance writers mostly work in collaboration on teams [8]. It remains an open question how and whether collaboration between multiple utterance writers would interact with the guidance provided in our tool, and should be further explored.

Finally, an important direction to explore in future research is applying the guidance principles that were tested here to user interactions with large language models. LLMs are initially built using enormous, broad-based language corpora, and then are subsequently fine-tuned with much smaller datasets consisting of context-specific language, which allows the LLM to provide relevant responses for individualized use cases or business purposes. To perform this fine-tuning, the model deployer needs to acquire or create a dataset of language that is tailored to their specific use case and topic. A clear extension of the current work is to use the feedback training tools from the current study to enable novices to create a unique dataset of utterances for fine-tuning an LLM for the deployer’s specific business context.

## 6. CONCLUSIONS

The study presented here demonstrates that novices can be guided to produce higher quality training utterances for a conversational agent with little start-up cost, by providing them with real-time, in-application linguistic feedback as they write. Both types of guidance, Linguistic Feedback and Sample Utterances, improved participants’ utterances along multiple dimensions which are often cited by experts as best practices to strive for, including heightened lexical diversity and richness, reduced syntactic similarity, and longer and more detailed utterances. The thresholds for such requirements could be easily adjusted for the specific model being trained and/or the policies of the organization training the model as every team has their own operational methods.

Providing this type of guidance could also be an effective way of training new utterance writers more rapidly, enabling teams to scale quickly and to have more content from subject matter experts in specialized domains. In addition, our methods could allow for crowd-sourcing utterances at scale, to substantially increase the breadth of the training set before a new agent is deployed. Thus, providing real-time linguistic guidance can empower novices to create quality training utterances for a new conversational agent, allowing for faster and more successful deployment.

## Figures and Tables

**Table 1:** The four intents employed in the experiment and two participant-written utterances for each.

Intent	Sample Utterances

Lost or Stolen Card	“My card has been pinched” “what do I do if I lost my card”
Compromised Card	“fraudulent activity on my card” “how can I block my card”
Card Arrival	“how long until I can use my new card” “i was supposed to get two cards but i only got one”
Order Physical Card	“new card delivery tracking code” “is there a fee for a new card?”

**Table 2:** The types of feedback shown in the Language Feedback condition in the tool. Note that “Term variety” and “Phrase variety” applied to all utterances as a whole, whereas the others were notifications that appeared at the level of an individual utterance when applicable.

Feedback Type	Description

Term variety	Level of similarity of words across all utterances.
Phrase variety	Level of similarity of syntactic structures across all utterances.
Similar terms	This utterance includes similar or related words to other utterances. For example, multiple utterances contain “lost card” and “stolen card.”
Similar phrasing	This utterance has a similar syntactic structure to other utterances. For example, “I need to return my product” and “I want to replace my card.”
Filler words	This utterance includes filler words which do not contribute content (e.g., “okay”, “please”, “thanks”).
Multiple intents possible	This utterance might contain multiple topics or requests to the conversational agent. This was determined by checking against a list that included words like “and,” “then,” and “after.”
Long utterance	This utterance exceeds the recommended length. The cutoff (17 words) was determined by selecting the 85th percentile of utterance length in the Banking77 [9] dataset.

## Data Availability

The data collected in this research (de-identified utterances, computed lexical features, and best practices features and notifications), as well as the code for running the statistical analyses, are publicly available on the Open Science Framework repository at https://osf.io/e9j8p/.

## CCS CONCEPTS

- Human-centered computing → Empirical studies in HCI.

## Footnotes

1
https://www.nltk.org/_modules/nltk/stem/porter.html

2
https://naturalnode.github.io/natural/brill_pos_tagger.html
